# Where are the values in evaluating palliative care? Learning from community-based palliative care provision

**DOI:** 10.1177/26323524241287223

**Published:** 2024-10-07

**Authors:** Sandy Whitelaw, Devi Vijay, David Clark

**Affiliations:** School of Social and Environmental Sustainability, University of Glasgow, Dumfries Campus, Bankend Road, Dumfries, DG1 4ZL, UK; Department of Organizational Behavior, Indian Institute of Management Calcutta, Kolkata, India; School of Social and Environmental Sustainability, University of Glasgow, Glasgow, UK

**Keywords:** community, complexity, evaluation, Kerala, India, palliative care, values

## Abstract

**Background:**

The World Health Organization Astana Declaration of 2018 sees primary healthcare as key to universal health coverage and gives further support to the goal of building sustainable models of community palliative care. Yet evaluating the benefits of such models continues to pose methodological and conceptual challenges.

**Objective:**

To explore evaluation issues associated with a community-based palliative care approach in Kerala, India.

**Design:**

An illuminative case study using a rapid evaluation methodology.

**Methodology:**

Qualitative interviews, documentary analysis and observations of home care and community organising.

**Results:**

We appraise a community palliative care programme in Kerala, India, using three linked ‘canvases’ of enquiry: (1) ‘complex’ multi-factorial community-based interventions and implications for evaluation; (2) ‘axiological’ orientations that foreground values in any evaluation process and (3) the status of evaluative evidence in postcolonial contexts. Three values underpinning the care process were significant: heterogeneity, voice and decentralisation. We identify ‘objects of interest’ related to first-, second- and third-order outcomes: (1) individuals and organisations; (2) unintended targets outside the core domain and (3) indirect, distal effects within and outside the domain.

**Conclusion:**

We show how evaluation of palliative care in complex community circumstances can be successfully accomplished when attending to the significance of community care values.

## Plain language summary

Where are the values in evaluating palliative care? Learning from community-based palliative care provision

The evaluation of any intervention or service will inevitably involve a series of decisions on what we measure, what criteria we use to judge whether the intervention has been successful (or not), what type of data we actually collect and what methods we use to do this. When evaluating a range of palliative care interventions, we suggest that these decisions have often been taken in a concealed way and tend to favour relatively narrow quantitative measures linked to end outcomes. Our paper reports on the evaluation of a community-based palliative care intervention on Kerala, India. In it, we suggest that such complex work requires a broader approach to evaluation that: makes the values being used to assess success explicit; draws on a range of data types; is interested in delivery processes; and places the voices of participants at the heart of the assessment. The paper concludes with some broader observations on how these principles might be applied more widely within palliative care.

## Introduction

In 2018, within the Declaration of Astana, the World Health Organization renewed its commitment to improving primary healthcare services and this, in turn, encouraged those committed to community-based palliative care provision.^1,2^ This form of practice can be seen as deriving the potential for care from civic resources.^3,4^ A question that follows concerns how such care provision is to be evaluated?^5^ Although progress has been made in evaluating the efficacy of a range of palliative care interventions at the micro level of individual patients and service users,^6–9^ we see less traction in tackling how to evaluate the more complex and multi-faceted forms of different palliative care service models.^10^ In this article and in the context of community palliative care provision, we use the ‘whole system’ Kerala community palliative care approach, founded on a networked model.

This network has been singled out as an exemplary example of its type by the Lancet Commission on the Value of Death,^11^ making it an appropriate case to explore the recognised challenges involved in evaluating any complex, multi-faceted community-based intervention (e.g. understanding the intricate relationship between processes and outcomes and negotiating the actual nature of such outcomes).^3^

Based on emerging theoretical and methodological pointers, community-related evaluation commentary focuses on three domains.^12,13^ Firstly, the delivery of palliative clinical and social care in community settings and at home.^14,15^ Secondly, wider forms of compassionate and caring community activity, expressed in relation to individual care, volunteer development and the promotion of caring communities.^16,17^ Thirdly, ‘whole system’ approaches that foster ‘joined-up’ networks of various community-based palliative care elements.^18,19^

Within such a wide range of practices, the definition of evaluative ‘objects of interest’ (the essential nature of the varied ‘outcomes’ that are being assessed) and any generalisation from them can be difficult.^20^ Although numerous examples exist, spanning processes and outcomes, drawing from quantitative and qualitative data, and relating to a variety of formalised professional and informal community contexts, most studies focus on specific areas, such as appraisal of community service delivery, mapping access to community palliative care, depicting networks between formal services and community and home resources, and estimating the capacity required to foster such networks, particularly relating to training programmes.^21,22^ Pain and symptom control, quality of life, cost outcomes, impact on secondary level services and public health literacy also feature as community palliative care-related objects of enquiry.^23–31^

Overall, efforts tend to be directed at outcome variables associated with individuals rather than the collective and ecological underpinnings and processes suggested by the notion of ‘community’.^32^ This reflects a tendency to focus on single objects of evaluative interest, at the expense of a broader system-based approach focussed on interdependent interactions that comprise complex community palliative care.^33^ This deficiency and the implications for evaluation strategies and design are beginning to be recognised,^34,35^ particularly in the wider social domain and related variables that are prominent in our chosen case study in Kerala.

Significant here is the progressive conceptual task of understanding profound *values* that inform our understanding of what might constitute a caring community and how we might evaluate it in the context of how care is valued in society and governed by situated ethical norms.^36–38^ These values are expressed in studies relating to community palliative care and include the exploration of the role of community at the end of life for people dying in advanced age, as seen from the perspective of bereaved family caregivers, as well as the complex delivery processes of community-based palliative care, and the processes that might define quality standards for community-based palliative care.^39,40^ For example, examining the Kerala community palliative care model surfaces four ‘logic conflicts’, reflecting competing values and frames of evaluative reference: (1) ‘professional’ and ‘community’ orientations; (2) ‘centralised’ and ‘decentralised’ governance; (3) ‘generalist’ and ‘specialist’ care and (4) ‘charity’ and ‘rights-based’ approaches.^41^ Moreover, any evaluation of Kerala’s community model must also be located within a postcolonial analysis that recognises and decentres the preponderance of West-centric values in assessing the quality of general and palliative care-specific interventions.^42,43^

### Our approach

Our study contributes to progressive forms of evaluation for three contextual canvases that we detail below. The complexity of the Kerala community model of palliative care has already been described, in which ‘community-led’ or ‘community-owned’ values promote egalitarianism, neighbourhood interdependence and solidarity.^42–47^ We build on these attributes in our approach to evaluation.

**Figure 1.**
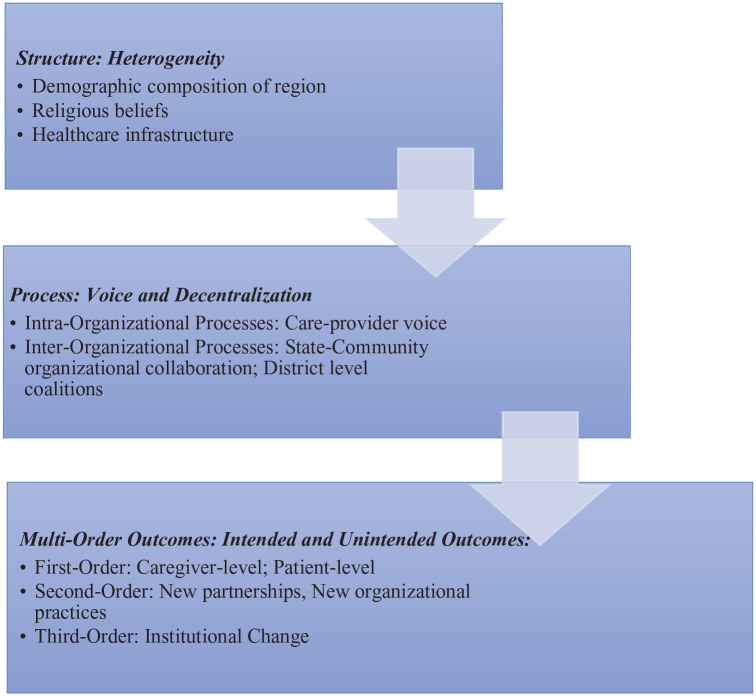
Adapted conceptual framework relevant to study.

There is growing acceptance of the inherent complexity at the heart of policy-making and implementation, resulting in a recognition that evaluative efforts require equivalent nuance.^3,48,49^ In applied terms and in contrast to traditional rationalistic ‘technical’ approaches, this understanding has led to the emergence of ‘interpretative’ policy analysis and a host of associated evaluative orientations that include realistic evaluation, deliberative methodologies, complexity cascade mechanisms, ‘practical rationality’ orientations and ‘judicial’ review models (Figure 1).^50–55^ Such evaluative lines allow us to gain access to the previously under-explored assumptions, values and implementation processes that articulate with various outputs, impacts and outcomes. We recognise the tendency for palliative care interventions to be evaluated based on outcome rather than process, and within that, to be largely devoid of any significant appraisal of values.^5^

In this context, our goal is to explore wider evaluation issues through an illuminative case study of the community-based palliative care approach in Kerala, India.^56^ The work arises from a recognition that, despite extensive affirmative literature on the proliferation of over 400 community palliative care organisations across Kerala^45^ and the problems that arise in translating the model to other states,^57^ evaluative accounts of the approach tend towards a weak ‘narrative’ orientation, specifically containing reductive and circular explanations of the success of the model, and particularly the centrality of opaque Keralite ‘cultural factors.’ Most accounts also lack an adequate account of their evaluation methodology.^58^

As a foundation, therefore, we first describe the social organisation of community palliative care in Kerala. We then outline the methodology that informed our work before presenting our results against three specific ‘canvases’:

(1) an interest in the evaluation of complex, multi-factorial community interventions within palliative care and their associated subtleties and intricacies^59^;
(2) axiological (i.e. ethical and aesthetic ‘value’) orientations that foreground the under-represented significance of the context^60^ that shape the delivery of such interventions, and the associated emergence of values-informed evaluation^61^ along with the formation and appraisal of various potential objects of interest^62^;
(3) the postcolonial context of enquiry^63^ in which frameworks developed in the Global North become benchmarks in assessing service outcomes in the Global South.^43^

We conclude by outlining a conceptual framework for the implementation of community-oriented evaluation exercises. In so doing, we advance the possibility that such empirical insights from the Global South can be theoretically telling beyond their particular context.^63^

### Kerala’s community palliative care model

Community-based approaches are a preferred global policy option for low-cost, resource-effective palliative care service provision.^64^ In this context, the model that emerged in northern Kerala in the late 1990s has become a global exemplar of a community-oriented palliative care approach.^45^ Here, services are locally available, and more profoundly, communities have an integral role in the work itself – they ‘own’ palliative care.^65^ In response to continued advocacy by community organisations, the state introduced a comprehensive palliative care policy mandating palliative care at the primary, and gradually at the community and tertiary levels.

Structurally, the palliative care field in Kerala today comprises community organisations, state-funded primary health centres and tertiary hospitals with nurses and physicians trained in palliative care, along with private hospitals with palliative care practitioners.^41^ In particular, there is a ‘bottom-up’ involvement of community members, often with little or no clinical background. This expanded version of ‘total care’ includes financial, social, emotional, medical and bereavement support for a wide range of patient categories.^43^ It includes those with advanced cancer, HIV/AIDS, diseases of old age, mental health problems and chronic renal and respiratory conditions. Within this wide-ranging community model, nurses and auxiliary nurses become ‘anchors’ for the home care team, with doctors (a relatively scarce resource) providing secondary support on a needs basis as well as access to morphine at the primary health level.^46^

As noted, and despite broad affirmation within global palliative care discourse,^45^ we recognise that the Kerala community model remains under-evaluated.^57^ Periodic and spatially fragmented evaluative work has been based on rather traditional and formalised approaches. For example, one study examined patient-level outcomes such as pain relief, quality of life, emotional and social well-being for cancer patients.^66^ Another assessed access and utilisation of palliative care services by government and community organisations in one Kerala region.^67^ Demographic, medical, pain and psychological factors associated with patients who choose the Kerala model have been surveyed.^68^ Likewise, a situational analysis of the quality of palliative care services across India has been conducted that includes some community organisations.^69^ However, published accounts have tended to be of a descriptive narrative type, tracing the Kerala initiative’s evolution, framing shifts and offering lessons learned, with an absence of work that formally and critically evaluates the community foundations of the model.^44–46^

## Methodology

Our methodology was informed by a whole system evaluative ethos and the specific recognition of a community palliative care network comprising system *structures, processes* of care and varied patient *outcomes*.^17,18^ We employed relational and participatory approaches that sought to co-produce consensual shared meaning with a range of stakeholders, including citizens, and with attention to practical reasoning.^70–72^

We developed a conceptual model of the criteria that multiple stakeholders might consider significant for the effective delivery of community palliative care. Specifically, we attend to the circumstantial political and cultural values that inform the delivery of such interventions and delineate how they may serve as objects of interest in evaluation and appraisal. We employed a rapid evaluation methodology – a well-established pragmatically oriented evaluative approach that seeks to generate timely and functional insights into interventions.^73,74^ This study spanned 6 months of data collection in 2019, drawing on the rapid evaluation method. The second author had conducted ethnographic work with Kerala’s community palliative care organisations over multiple stages between 2009 and 2019, starting with her doctoral work and continued commitments thereafter. For example, she participated in national palliative care conferences and documented the translation of Kerala’s community palliative care initiative to West Bengal, thus gaining situated insights into the emergence and evolution of the palliative care field,^4,75,76^ structural factors^77^ and geographical differences in palliative care provision.^57^

For this study, all interviews and field visits were undertaken by a field team comprising the second author and three fieldwork assistants who were trained community palliative care volunteers. While the second author knew several long-standing community palliative care physicians, nurses and volunteers, the field assistants facilitated access to newer community organisations and with patients and families with whom they had established relationships.

This approach ensured that we purposively sampled for heterogeneous, sometimes conflicting views, from geographically dispersed locations. Simultaneously, working with community volunteers enabled us to mitigate the intrusive empiricism that could arise when the lives of those suffering are opened up for scrutiny, and voyeuristic revelations during research processes.^78^

We interviewed care providers in their offices, clinics or common rooms at the workplace. We typically met with patients and families at their homes. We accompanied community organisations for home care visits. The second author informed all study participants about the objective of evaluating community palliative care provision. The interviews followed a conversational style, building on an interview protocol adopted by the team that guided conversations around evaluating quality. In some cases, particularly with care providers who had worked for decades, we had follow-up conversations to clarify elements we discerned from other interviews. The entire field team engaged in daily debriefs. The research team met face-to-face twice during this period to discuss emergent findings and presented the preliminary findings to an interdisciplinary End-of-Life Care research group for additional inputs. Finally, this work is also informed by the research team’s engagement with palliative care over a long period. The first author (SW) brought specific insights and experience around the variety of approaches to evaluation. The second author (DV) has had experience working with community-based palliative care services in Kerala and conducted the fieldwork and data analysis. The third author (DC) has had a long-term, extensive role in researching the history, evaluation and global development of palliative care services and models.

Our methods are summarised in Table 1 below.

**Table 1.** Summary of project.

Method	Empirical work
In-depth qualitative interviews	11 patients, 3 family members, 12 physicians, 8 nurses, 17 volunteers, 6 paid staff, 2 community mental health professionals who work with community palliative care networks, 1 government officials, 1 state elected representative and 1 journalist Average interview length: 45 min
Documentary analysis	Relevant palliative care policy documents, media articles and scientific articles
Observations of home care and community organisational functioning	Field notes on visits to nine clinics sampled based on geographic dispersion across North, South and Central Kerala

## Results

Consistent with a realistic evaluative whole system approach, we organise our findings based on notions of structure, processes and multiple forms of outcomes.^17^

### Context of community care: Valuing heterogeneity

The over-arching context can be understood in relation to a series of features related to basic demography and workforce composition, religious beliefs and healthcare infrastructure.

#### Demographic and workforce composition features

Demographic and workforce composition were two structural elements that shaped patient-level outcomes, caregiver mobilisation and inter-organisational processes. Demographic parameters such as caste, religion, gender and rural (vs urban) distributions were significant structural factors as vectors of service accessibility, and indicators of disparities in access and exclusions. For example, caste markers of purity and pollution shape healthcare provision in India, with specific inflections in Kerala, wherein specific groups are disproportionately engaged in the embodied work of nursing^74^ while oppressed castes and tribes continue to face exclusions in healthcare provision.^79,80^ In our field observations, community caregivers were from diverse caste groups, and we did not observe incidents of resistance to home care provision based on caste identities. However, in interviews, participants shared how caste identity can be an impediment to translating community palliative care in other geographies, such as Puducherry (a separate Union Territory; also highlighted in a recent study^81^). Therefore, a systematic effort to evaluate marginalisation across geographies even in Kerala is necessary, given the entrenched, ‘invisibilised’ and caste-blind ways in which caste vectors operate.^82,83^

Similarly, nurses who anchor home-based care provision, tend predominantly to be women. On the one hand, nurses reported in interviews about the more ‘empowering’ and humane character of community palliative care provision that re-centred patients and reinforced for them the reasons they chose nursing as a profession. On the other hand, nurses continue to be undervalued and underpaid as care workers,^74^ and relegated to a secondary role in decision-making, indicating a gendered care burden that needs specific attention. A physician who worked towards building the community mental health initiative in close collaboration with the palliative care networks considered that community palliative care had ‘democratised care’. However, he observed that prevailing patriarchal structures continued to shape attitudes towards women who were crucially involved in service provision:

Initially, I thought that would lead to a breaking away of existing structures but what has happened is men holding power locally, they become interested in their own traditional ways.

While it is beyond the ambit of this study to examine how patriarchal power structures shaped palliative care delivery in different organisations, such accounts suggest the need for attention when evaluating community ownership and participation.

#### Religious beliefs

Community organisations in our dataset do not explicitly identify as faith-based, and care providers emphasised that ‘palliative care should not have religion’ and ‘religion should not be brought into palliative care’. Nevertheless, religion played a palpable role in service delivery. Kerala is religiously diverse, with 55% Hindus, 26% Muslims, 18% Christians and the role of religion as an institutional structure is considered significant across each of these faiths.^84^ Religious practices such as *zakat*, or the mandatory donation to charity in Islamic beliefs, are routinely mobilised to support palliative care. Also, faith-based organisations serve as established mobilising structures that facilitate the recruitment of volunteers and undertake fund-raising and awareness campaigns. Some participants suggested that their religious beliefs motivated and sustained their care practices. For example, Ashraf a student volunteer in his mid-20s who was completing his Master’s degree in Social Work, informed us that he was introduced to palliative care through a socio-religious organisation that his parents and brother also worked with. He reflected that while his entry into community volunteering was because of his family’s religious beliefs, working in palliative care had made him less religious: ‘I used to pray five times a day. I still do it here, but here all are communists, atheists. I am influenced. Now, it is like doing social work, without being too religious’. Such reflections shed light on how religious beliefs intersect with political ideologies and social work commitments during care provision.

#### Healthcare infrastructure

‘Health infrastructure’ reflected the availability of medical professionals, public and private hospitals, community and primary health centres. Compared to the rest of India, there are relatively more hospitals in Kerala with more geographical accessibility.^85^ Kerala has a relatively high density of trained medical professionals, including physicians and nurses.^77^ Furthermore, the spending on public health services has had a ‘crowding-in’ effect, as the competition between public and private delivery services has led to increased overall efficiency.^86^ However, public health infrastructural access and affordability have been weakened by increasing levels of privatisation across India generally and Kerala particularly.^87^

Specifically, in terms of community palliative care infrastructures, there are variations across the state. For instance, Kozhikode city has two active community palliative care initiatives providing 24/7 support, with the government hospital providing an additional home care service for its patients. In contrast, a community organisation in a small town in Wayanad relies on a physician who is already serving at a primary health centre in another town, and who comes to the clinic for patient consultations after finishing his daily roster of work.

Our interviews and observations also highlighted variations in training levels. For example, in one remote clinic that we visited, only one of four nurses was trained in palliative care. The physician explained that a trained palliative care nurse made a difference even with basic procedures like catheterisation, which a palliative care nurse performed several times more per month than a generalist nurse.

In summary, while the Kerala model’s (unevaluated) efficacy has been attributed to ‘cultural’ factors, we found these explanations inadequate and essentialist. The heterogeneous demographic and workforce composition, religious beliefs and variations in healthcare infrastructures do not adequately substantiate a monolithic explanation. In contrast to care models shaped by professional worth, determined by medical professionals, or economic models that underpin a logic of market-efficient or financially viable health infrastructures, the community model is premised on securing coordination among heterogeneous social groups that shape care provision. This heterogeneity which sustains the model, complicates one-dimensional evaluation indicators to assess standards and quality of care provision. Our observations call for systematic analysis of variations that point to basic principles that can be adopted by community organisations.

### Process of community care: Valuing voice and decentralisation

Complementing the structural influences, voice and decentralisation were two features relating to the *process* of care that underpinned *intra*-organisational and *inter*-organisational processes.

#### Voice

Our interlocutors repeatedly shared how they valued having a voice for independent decision-making in *intra-*organisational processes. For example, the volunteers’ role has been prominent in the community model. An early tenet of the community model was that ‘anyone can be a volunteer’.^88^ Some long-standing volunteers are from working-class or lower-middle class backgrounds – outsiders to the medical domain^75^ and undertake a range of tasks, from surveying households for patients, following up with patients and families in their neighbourhoods, spending time with a patient if the primary caregiver was not at home, to assisting patients in their wheelchair.

These volunteers emphasised the importance of having a ‘voice’ within their voluntary work. They appreciated a flatter organisation structure that created space for their perspectives, being listened to and given genuine responsibilities. For example, a paid employee with a pioneering palliative care organisation highlighted that, ‘the way they behave with us, how other doctors relate with us. . .they behave like they are one among us. . .all are equal here’. Similarly, nurses reported significant flexibility and meaning in their work. A palliative care nurse contrasted her experience with her public hospital nursing experience as follows: ‘in a hospital setting, we give patients what they need. In the hospital, doctors write, we look at the case sheet and follow what needs to be done. There is no attachment with the patients’. In contrast, with community palliative care, she felt: ‘home-care it is different, we can do our duty in home care. We are attached to patients. There is a bond, and we gain satisfaction’.

#### Decentralisation

We also traced *inter*-organisational collaborations *between* community organisations, local self-governing institutions and state-funded primary health centres. Public-community collaborations were decentralised to the district level (e.g. Ernakulam), or sub-district level (e.g. Malappuram) and varied in their nature. For example, in some regions like Nilambur and Kozhikode, the community organisations and primary and tertiary government health centres had devised collaborations – dividing the region zonally to avoid redundancies, mitigate turf battles and ensure continuity of care. In some regions, we observed fluid boundaries between state and community organisations, with state physicians and/or nurses serving extra time at local community organisations, or explicit partnerships between these two organisational actors.

In the regions where the collaborations worked, the stakeholders emphasised how public infrastructures could address the issue of resource mobilisation (funds and personnel) that community organisations grappled with. Problematically, a few stakeholders pointed out that in some regions, state-funded primary health centres are relatively less accessible and inactive, the work done superficially to ‘tick the box’. This was seen to result in community organisations stepping up meeting needs unfulfilled by the state and more widely, bridging an institutional vacuum of state-funded primary or district hospitals.

In summary, in this *process* domain, we see that care delivery was valuable for different stakeholders, not just in terms of whether care is provided, but how much *voice* they have in making and tinkering with care provision.^76^ Additionally, member voice is significantly enabled by decentralised forms of organising.

### Multi-order outcomes of community care: Valuing intended and unintended consequences

Above, we established the possibility of a wide range of potential evaluative ‘objects of interest’ associated with complex community interventions. Here, we identify such variation in relation to what we identified as first-, second- and third-order field outcomes.

#### First-order outcomes

These first-order outcomes related to both individuals *and* organisations and were intended direct effects of organising (e.g. clinical outcomes for patients, care-provider satisfaction) that arise for the intended targets (e.g. patients, care providers). In the community model, these were associated with patients *and* carers and volunteers. Regarding patient outcomes, we found wide differences in patients’ expectations and needs within community services. Patients from agricultural/working-class backgrounds consistently appreciated the free medicines and availability of medical care and medicines at the doorstep.^4^ This is salient in the context of the Indian healthcare system, where getting access to a doctor in a public hospital can be difficult. Moreover, a bystander would have to accompany a patient, or go alone to fetch medicines, resulting in loss of day wages. In these circumstances, the biggest relief for low-income families is the free medicines provided by community organisations and primary health centres. With the government’s involvement in primary healthcare-level palliative care, the monthly check-up by the nurse and medicine provision was a respite for the family. While middle-class patients valued the nurse and volunteer home care visits, often forming strong ties and camaraderie with the visiting team, they expressed appreciation for access to qualified expert physicians.

Furthermore, outcome benefits accrued to volunteer caregivers, who articulated an increased sense of meaning, dignity and social capital regarding access to networks and career opportunities, with some volunteers getting trained as palliative care nurses and taking on administrative roles in associated NGOs. For example, Shabnam, a quadriplegic patient from an affluent family, found meaning in being a ‘volunteer’, rather than being seen as a patient. Shabnam’s family is affluent and has high social and cultural capital in Kozhikode City. When her father first told her about a residential rehabilitation and vocational training workshop for paraplegic and quadriplegic patients, Shabnam was dismissive, since she had never stayed away from home. Unlike other patients who wanted to learn skills for a livelihood, she had no constraints. However, Shabnam has been a volunteer now for 9 years. Here, we do not infer that parameters like social inclusion and meaningful engagement were unimportant for low-income and middle-class patients and families. Rather, the more salient issues and expectations centred around material constraints to medicines, access to resources and so on. Thus, we see the distinct ways in which patients from different class categories relate to the palliative care service that offered them voice.

Notably, beyond the realm of calculated actions, Varman et al.^4^ also identified agapic love as a desirable unintended consequence of such transformative care models. Building on Boltanski’s^89^ conceptualisation of agape, we note that while care providers may have diverse values that underpin their calculation of justice in caregiving situations, in certain situations, care providers act selflessly out of love, with no expectations of reciprocity or instrumental returns. Indeed, M. R. Rajagopal, a pioneer of palliative care in Kerala, has stated that:

Many of us have a selfish kind of love. . . In the process, we push for useless and inappropriate treatment that increases and prolongs suffering. A deeper, more humane love is to recognize the suffering and accept the incurability; accept the inevitability of death. Compassionate care facilitates quality of life for as long as possible, and then facilitates quality of death – a good death, that is free of suffering as much as possible, while receiving and giving love.^89^

Statements like this underscore the under-recognised role of love in care settings.

#### Second-order outcomes

These second-order outcomes were seen to impact individuals or organisations as unintended targets outside the original domain and were expressed in a variety of ways by our participants. Firstly, involvement in community palliative care was considered to have enhanced capacity that comes from the pooling of expertise and resources and the fostering of a new, innovative initiative. For example, we identified a range of new interventions (e.g. ‘Students’ Initiative in Palliative Care’) that had gained momentum. Some organisations established death cafes where members met to discuss bereavement or their experiences of caregiving. Moreover, community organisations embraced new patient categories, such as chronic renal conditions and associated dialysis services. Furthermore, the community mental health movement in Kerala emerged from the palliative care movement, with community volunteers including mental health in palliative care services. The spin-off independent community mental health initiatives now draw on palliative care networks in north and central Kerala.

Collaboration was seen to result in ‘second-order’ benefits in the socio-political sphere. Community organisations made claims for public health integration resulting in a state-level palliative care policy mandating palliative care at the panchayat (village councils of elected representatives) level. Close collaboration between palliative care organisations and *panchayats* has resulted in a policy whereby *panchayats* must apportion a percentage of their budget for palliative care projects in their annual proposals. An elected representative and previous state government minister who was a proponent of the palliative care movement in his constituency talked to us about plans to further embed palliative care into primary health systems, whereby primary health centres could become family wellness centres, addressing prevention, treatment and palliative care rehabilitation. He identified the crucial role of community, participatory movements, especially in prevention and palliative care modules. In a similar political vein, the Communist Party of India (Marxist) (CPI(M), currently the ruling coalition in Kerala), has trained party cadres in palliative care. While the CPI (M) does not run a community organisation directly, this initiative is a measure to increase the CPI(M)’s grassroots presence. Moreover, there is a long-term anticipated gain of social prestige in terms of engagement in terminal and chronic care issues.

#### Third-order outcomes

These were indirect, distal effects both within and outside the core domain. For example, government initiatives anchored around preventive approaches to address at-risk adolescents, were spearheaded by an active palliative care volunteer and drew on a palliative care volunteer network. While there is no overlap in the functioning of the two initiatives, such instances exemplify the cascading and spill-over effects of social mobilisation. Similarly, individuals volunteering in palliative care have started NGOs in unrelated domains, such as rehabilitation efforts for differently abled individuals. Similarly, during the 2017–2018 floods in Kerala, 2018 Nipah outbreak and during the COVID-19 pandemic, community palliative care networks were mobilised for care.^90^

To summarise, in examining these outcomes, we learn to ask where we must locate evaluation, ‘in the object of appreciation or the appreciating subject’.^75^ We see that care delivery acquires different values for people from different classes and has second-order and distal effects for unintended targets.

## Discussion

At the onset, we described existing efforts to evaluate community-based palliative care and set up three canvases against which such work can be appraised. We return to these canvases now in light of our empirical insights. Our case exists in a wider debate about how to optimally evaluate palliative care and create and interpret the associated notion of ‘evidence’.^91^ Despite the discipline’s ability to deviate in theory and practice from the biomedical orthodoxies that shape other clinical specialisms,^11^ when it comes to evaluation, it appears to still find itself entangled in objectivist and economistic evidence-based medicine doctrines.^92^ For example, there is a tendency to focus on isolated tangible outcomes such as ‘pain’ and ‘symptom control’^93^ and to apply rudimentary cost-effectiveness analyses.^94^ In applying strict ‘Cochrane-based’ principles to these measures, there is ‘a dearth of good quality primary studies in the field of palliative care’ and ‘weak evidence of the benefits of any intervention’^95^ along with inconclusive evidence on cost-effectiveness.^94^ Consequently, some hold the view that palliative care researchers have ‘lagged significantly behind their counterparts in their development and use of evidence-based medicine (EBM)’.^92^

Others have countered this pessimism by contending that palliative care is a ‘complex multi-faceted public health intervention’ and that simple hierarchically based, outcome-focussed objects of interest are ‘too rigid and inappropriate’.^96^ Keeping with an evolution from ‘evidence-based *medicine*’^97^ emphasising solely the outcomes of 1-1 clinical interventions to ‘evidence-based *public health*’,^98^ our work suggests that an alternative evaluative environment is possible.

**Figure 2.**
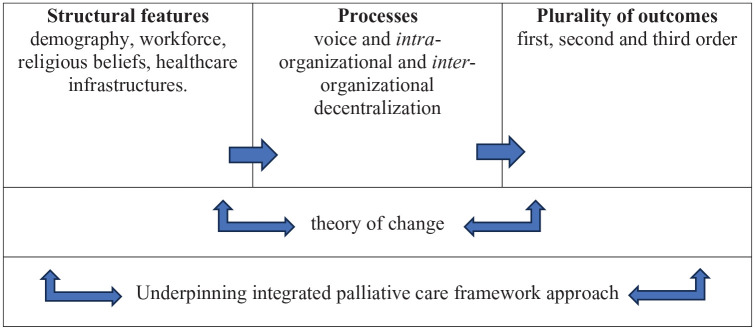
An evaluative system.

Firstly, we shift the framing of the evaluative domain from single isolated ‘interventions’ to the potential of multiple ‘events’ within a systems orientation.^99,100^ Additionally, in utilising an ‘integrated palliative care framework’,^17^ we explicitly draw on ‘theory of change’^101^ resources in seeking to understand the mechanisms underlying Kerala’s community model. Finally, with respect to outcomes, we allow the identification of a *plurality* of (first, second and third order) manifestations. This ground is summarised in Figure 2 below.

In developing a conceptual framework for evaluation of the community model of palliative care, we also underscore *values* as key and constitutive features. In relation to the broad conduct of the work, we foreground relational and participatory approaches with a wide range of stakeholders that foster consensual ‘co-production’. Specifically, we surface the heterogeneity of values at play, the need for voice and decentralised collaborations to enable those articulating these heterogeneous values, and the intended and unintended associations between the evaluative object and the subjects of evaluation. An extensive survey of palliative care provision across India which included public and community services in Kerala, usefully specifies multiple ‘essential criteria’.^70^ Here, the authors check for parameters such as ‘evidence of interaction between community and healthcare professionals in the establishment and ongoing operations of services’. However, heterogeneity, voice and decentralisation are not entirely captured in these ‘interactions’ and appear in our study as crucial values constitutive of community care. In effect, evaluative exercises stand to benefit from a focused analysis of values that matter to stakeholders and are constitutive of a deliberative, participatory system that mutualises knowledge.

We round-off this discussion by reflecting on this evaluative process for a community model in a postcolonial context. Bioethical norms continue to ‘originate in the Global North and “diffuse” and globally overdetermine ethical fields’.^102^ We challenge ‘the dominant ways through which the world is known and how this knowledge is defined’ and consequently, ‘a Western intellectualism and knowledge hegemony’^103^ that creates a monoculture in the global health domain.^104^ Zaman et al.^43^ have specifically critiqued the West-centric vision of ‘one joint future of end-of-life care in contrast to the Kerala community model, which demonstrates ‘how with the support of the specialists, “community assets” can be the main “logistics” to achieve a good quality death for large numbers’.^43^ The notion of a ‘culturally competent, appropriate or responsive’ approach to evaluation^63^ with first nation perspectives foregrounded is established within a context of a ‘decolonising’ ethic – ‘a systematic way of research and evaluation that attempts to liberate the colonized mind’.^105^ Such influences have been profound in our thinking about how we undertake evaluation.^42^

Our approach here is synonymous with these principles and seeks to unsettle hegemonic knowledge-flattening projects that locate palliative care benchmarks in the Global North. We explicitly recognise the political context and demographic features that shape Kerala’s community model – we incorporate as wide a range of stakeholder views as possible and particularly foreground situated community cultures and values that shape perceptions of the community model’s worth grounded in the warp and weft of location. This latter theme is a prominent feature of a decolonised approach to evaluation where the ‘axiology’ orientation prioritises perspectives that decentre West-centric positions as well as fostering class, caste, religious and ethnic privileges,^42^ particularly those that are ‘grounded on collective responsibilities, cooperation, interdependence, and interpersonal relationships’ and deviate from ‘Euro-Western research paradigms (that) reinforce blind reliance on Eurocentric models, strategies, and techniques’.^63^

## Concluding considerations

In conclusion, we offer broader reflections from our experiences. Firstly, our work provides a detailed case study of the practicalities of undertaking the evaluation of palliative care in complex community circumstances. It shows that, with a sensitive appreciation of structural features, a strong theoretical framework to explore delivery and a set of associated methods, then multiple and balanced insights can be gained. In particular, we highlight an ‘axiological’ orientation – the significance of context-specific values in undertaking research and providing a lens through which evaluative ‘worth’ is established. Dominant Eurocentric research and evaluation paradigms assume an epistemic, axiological and ethico-political universality by excluding such expressions of different forms of worth. The alternative offers the possibility of a more sophisticated and decolonised evaluation of community palliative care interventions. In this context, we develop and extend the pluralistic community palliative care evaluative approach that balances emphases on processes and outcomes, qualitative and quantitative data and the possibility of a variety of evaluative objects of interest.^106^ These technical developments are also suggestive of deeper foundations – for example, the possibility of a ‘post-normal’ evaluative domain^73^ where insight is not sought through quantitative data and reductionism, but rather by embracing interpretive social science.^107^

This ground is also suggestive of politically driven influences. We have highlighted the significance of ‘axiological’ perspectives in grounded evaluation. We suggest that these tend to be suppressed or hidden in the objectivist approaches embodied in the evaluation of individual interventions and, more broadly, in the construction of ‘league tables’ and ‘world maps’.^108^ By accentuating values, evaluation has the potential to become a set of ongoing incremental social and political processes where complexity and difference in perspectives are accepted and embraced.^99^ The consequences of this stance are a preference for localised^109^ and ‘mosaic’^110^ collective health orientations towards evidence. This ability to deviate from orthodoxies is also embodied in a more profound ‘decolonial solution’ which offers the potential for ‘more context-sensitive, responsive, representative and participatory research or evaluation design’.^111^

Some may see an axiological perspective as problematic – a capitulation to subjectivity and localism whilst giving up hope of achieving the objective ‘one joint future’ consensus of ‘evidence-based’ palliative care. Our contention is that, whilst such aspirations and their associated claims to rational rigour are undeniably attractive and may be one way of approaching evidence, they cannot be the only way. Furthermore, in the widely accepted complex and heterogeneous nature of palliative care,^8^ these one-dimensional strategies can be relatively rudimentary and potentially lead us to cling uncritically to a superficial sense of objectivity. Simultaneously, the realism and plurality of value-based evaluation informed by postcolonial contexts cannot be mere ‘add-ons’ and ‘toppings’, but can fundamentally re-constitute the composition of what we consider viable, realistic palliative care evidence.

## Declarations

**Ethics approval and consent to participate:** Approval provided from Indian Institute of Management Calcutta. Technical partner for this study was the Institute of Palliative Medicine Kozhikode.

**Consent for publication:** Not applicable.

**Availability of data and materials:** The authors can be contacted for the database of documentary data. The primary interviews are masked based on confidentiality and anonymity.

## References

[1] World Health Organization. Declaration of Astana: global conference on primary health care: Astana, Kazakhstan, 25 and 26 October 2018 (No. WHO/HIS/SDS/2018.61). Astana, Kazakhstan: World Health Organization, 2019.

[2] Pettus K Moine S Kunirova G , et al. Palliative care comes of age in the 2018 Declaration of Astana. J Palliat Med 2019; 22(3): 242. 30794491 10.1089/jpm.2018.0615

[3] Skivington K Matthews L Simpson SA , et al. A new framework for developing and evaluating complex interventions: update of Medical Research Council guidance. BMJ 2021; 374: 2061. 10.1136/bmj.n2061 PMC8482308 34593508

[4] Varman R Vijay D Skålén P . The conflicting conventions of care: transformative service as justice and agape. J Serv Res 2022; 25(1): 86–107.

[5] Vernon E Hughes MC Kowalczyk M . Measuring effectiveness in community-based palliative care programs: a systematic review. Soc Sci Med 2022; 296: 114731. 35131612 10.1016/j.socscimed.2022.114731

[6] Kaasa S Forbes K . Research in palliative care. In: Cherny NI Fallon MT Kaasa S , et al. (eds) Oxford textbook of palliative medicine. Oxford: Oxford University Press, 2021, pp. 1261–1267.

[7] Farrar JT . Understanding clinical trials in palliative care research. In: Cherny NI Fallon MT Kaasa S , et al. (eds) Oxford textbook of palliative medicine. Oxford: Oxford University Press, 2015, pp. 1167–1175.

[8] Kaasa S Håvard Loge JH . Quality of life in palliative care: principles and practice. In: Cherny NI Fallon MT Kaasa S , et al. (eds) Oxford textbook of palliative medicine. Oxford: Oxford University Press, 2015, pp.1198–1209.

[9] Mathew C Hsu AT Prentice M , et al. Economic evaluations of palliative care models: a systematic review. Palliat Med 2020; 34(1): 69–82. 31854213 10.1177/0269216319875906

[10] Vanderstichelen S Deliens L . Complexities and challenges in public health palliative care research. In: Abel J Kellehear A (ed.) Oxford textbook of public health palliative care. Oxford: Oxford University Press, 2022, pp. 245–254.

[11] Sallnow L Smith R Ahmedzai SH , et al. Report of the Lancet Commission on the Value of Death: bringing death back into life. Lancet 2022; 399: 837–884. 35114146 10.1016/S0140-6736(21)02314-X PMC8803389

[12] Vanderstichelen S Dury S De Gieter S , et al. Researching compassionate communities from an interdisciplinary perspective: the case of the compassionate communities center of expertise. Gerontologist 2022; 62(10): 1392–1401. 35263765 10.1093/geront/gnac034

[13] Abel J Kellehear A Karapliagou A . Palliative care – the new essentials. Ann Palliat Med 2018; 7(Suppl. 2): S3–S14. 10.21037/apm.2018.03.04 29764169

[14] Farr M Barker R . Can staff be supported to deliver compassionate care through implementing Schwartz Rounds in community and mental health services? Qual Health Res 2017; 27(11): 1652–1663. 28799475 10.1177/1049732317702101

[15] Koski J Kelley ML Nadin S , et al. An analysis of journey mapping to create a palliative care pathway in a Canadian First Nations community: implications for service integration and policy development. Palliat Care 2017; 10: 1178224217719441. 28794638 10.1177/1178224217719441 PMC5524242

[16] Dodd S Hill M Ockenden N , et al. ‘Being with’ or ‘doing for’? How the role of an end-of-life volunteer befriender can impact patient wellbeing: interviews from a multiple qualitative case study (ELSA). Support Care Cancer 2018; 26(9): 3163–3172. 29594487 10.1007/s00520-018-4169-2 PMC6096531

[17] Bollig G Brandt F Ciurlionis M , et al. Last Aid course. An education for all citizens and an ingredient of compassionate communities. Healthcare 2019;7(1): 19. 30696095 10.3390/healthcare7010019 PMC6473377

[18] Bainbridge D Brazil K Ploeg J , et al. Measuring healthcare integration: Operationalization of a framework for a systems evaluation of palliative care structures, processes, and outcomes. Palliat Med 2016; 30(6): 567–579. 26934948 10.1177/0269216315619862

[19] Bainbridge D Brazil K Krueger P , et al. A proposed systems approach to the evaluation of integrated palliative care. BMC Palliat Care 2010; 9(1): 8. 20459734 10.1186/1472-684X-9-8 PMC2876145

[20] Dumont K Marcoux I Warren É , et al. How compassionate communities are implemented and evaluated in practice: a scoping review. BMC Palliat Care 2022; 21(1): 131. 35854292 10.1186/s12904-022-01021-3 PMC9297657

[21] Avis M Jackson JG Cox K , et al. Evaluation of a project providing community palliative care support to nursing homes. Health Soc Care Community 1999; 7(1): 32–38. 11560620 10.1046/j.1365-2524.1999.00158.x

[22] Horsfall D . Developing compassionate communities in Australia through collective caregiving: a qualitative study exploring network-centred care and the role of the end of life sector. Ann Palliat Med 2018; 7(S2): S42–S51. 10.21037/apm.2018.03.14 29764172

[23] Grindrod A Rumbold B . Providing end-of-life care in disability community living services: an organizational capacity-building model using a public health approach. J Appl Res Intellect Disabil 2017; 30(6): 1125–1137. 28544672 10.1111/jar.12372

[24] Gardner DS Doherty M Ghesquiere A , et al. Palliative care for case managers: Building capacity to extend community-based palliative care to underserved older adults. Gerontol Geriatr Educ 2022; 43(2): 269–284. 30442079 10.1080/02701960.2018.1544129

[25] Eagar K Clapham SP Allingham SF . Palliative care is effective: but hospital symptom outcomes superior. BMJ Support Palliat Care 2020; 10(2): 186–190. 10.1136/bmjspcare-2018-001534 PMC7286033 30171042

[26] Potts M Cartmell KB Nemeth L , et al. A systematic review of palliative care intervention outcomes and outcome measures in low-resource countries. J Pain Symptom Manage 2018; 55(5): 1382–1397. 29305322 10.1016/j.jpainsymman.2017.12.487

[27] Newbury J . Symptom control outcomes in a community palliative care nursing team. Int J Palliat Nurs 2002; 8(1): 6–12. 11823744 10.12968/ijpn.2002.8.1.10237

[28] Choi S-O Kim S-N Shin S-H , et al. Evaluation of outcomes of the Busan Community-based Palliative Care Project in Korea. Asian Nurs Res (Korean Soc Nurs Sci). 2018; 12 (4): 286–92. 10.1016/j.anr.2018.11.001 30448262

[29] Yosick L Crook RE Gatto M , et al. Effects of a population health community-based palliative care program on cost and utilization. J Palliat Med 2019; 22(9): 1075–1081. 30950679 10.1089/jpm.2018.0489 PMC6735317

[30] Kim SH Chung BY Xu Y . Evaluation of a home-based hospice and palliative care program in a community health center in Korea. Asian Nurs Res (Korean Soc Nurs Sci) 2009; 3(1): 24–30. 25030229 10.1016/S1976-1317(09)60013-X

[31] Seymour J . The impact of public health awareness campaigns on the awareness and quality of palliative care. J Palliat Med 2018; 21(S1): S30–S36. 10.1089/jpm.2017.0391 PMC5733664 29283867

[32] Librada-Flores S Nabal-Vicuña M Forero-Vega D , et al. Implementation models of Compassionate Communities and compassionate cities at the end of life: a systematic review. Int J Environ Res Public Health 2020; 17(17): 6271. 32872244 10.3390/ijerph17176271 PMC7504622

[33] Otis-Green S Yang E . ACE project – advocating for clinical excellence: creating change in the delivery of palliative care. Omega 2013; 67(1–2): 5–19. 23977775 10.2190/OM.67.1-2.b PMC3826786

[34] Hazelwood MA Patterson RM . Scotland’s public health palliative care alliance. Ann Palliat Med 2018; 7(Suppl. 2): S99–S108. 10.21037/apm.2018.03.13 29764176

[35] Pfaff KA Dolovich L Howard M , et al. Unpacking ‘the cloud’: a framework for implementing public health approaches to palliative care. Health Promot Int 2020; 35(1): 160–170. 30690474 10.1093/heapro/day123

[36] Wegleitner K Schuchter P . Caring communities as collective learning process: findings and lessons learned from a participatory research project in Austria. Ann Palliat Med 2018; 7(Suppl. 2): S84–S98. 10.21037/apm.2018.03.05 29764175

[37] Sayer A . Why things matter to people: social science, values and ethical life. Cambridge: Cambridge University Press, 2011.

[38] Jennings B . Solidarity and care as relational practices. Bioethics 2018; 32(9): 553–561. 30264873 10.1111/bioe.12510

[39] Gott M Wiles J Moeke-Maxwell T , et al. What is the role of community at the end of life for people dying in advanced age? A qualitative study with bereaved family carers. Palliat Med 2018; 32(1): 268–275. 29130405 10.1177/0269216317735248

[40] Mistry B Bainbridge D Bryant D , et al. What matters most for end-of-life care? Perspectives from community-based palliative care providers and administrators. BMJ Open 2015; 5(6): e007492. 10.1136/bmjopen-2014-007492 PMC4486948 26124510

[41] Vijay D Whitelaw S Clark D . Logic conflicts in community-based palliative care. Prog Palliat Care 2021; 29(3): 149–155.

[42] Chilisa B Major TE Gaotlhobogwe M , et al. Decolonizing and indigenizing evaluation practice in Africa: toward African relational evaluation approaches. Can J Program Eval 2016; 30(3): 313–328.

[43] Zaman S Inbadas H Whitelaw A , et al. Common or multiple futures for end of life care around the world? Ideas from the ‘waiting room of history’. Soc Sci Med 2017; 172: 72–79. 27894008 10.1016/j.socscimed.2016.11.012 PMC5224187

[44] Kumar S . Models of delivering palliative and end-of-life care in India. Curr Opin Support Palliat Care 2013; 7(2): 216–222. 23635881 10.1097/SPC.0b013e3283610255

[45] Sallnow L Kumar S Numpeli M . Home-based palliative care in Kerala, India. Prog Palliat Care 2010; 18: 14–7.

[46] Kumar SK . Kerala, India: a regional community-based palliative care model. J Pain Symptom Manage 2007; 33(5): 623–627. 17482058 10.1016/j.jpainsymman.2007.02.005

[47] Vijay D Kulkarni M . Frame changes in social movements: a case study. Public Manag Rev 2012; 14(6): 747–770.

[48] Tenbensel T . Complexity in health and health care systems. Soc Sci Med 2013; 93: 181–184. 23845660 10.1016/j.socscimed.2013.06.017

[49] May C Johnson M Finch T . Implementation, context and complexity. Implementation Sci 2016: 11: 141. 10.1186/s13012-016-0506-3 PMC5069794 27756414

[50] Browne J Coffey B Cook K , et al. A guide to policy analysis as a research method. Health Promot Int 2019; 34(5): 1032–1044. 30101276 10.1093/heapro/day052

[51] Pawson R Tilley N . Realistic evaluation. London: Sage, 1997.

[52] Dahler-Larsen P . Can we use deliberation to change evaluation systems? How an advisory group contributed to policy change. Evaluation. 2023; 29(2): 144–160.

[53] Geyer R . Can complexity move UK policy beyond ‘evidence-based policy making’ and the ‘audit culture’? Applying a ‘complexity cascade’ to education and health policy. Polit Stud 2012; 60(1): 20–43.

[54] Sanderson I . Complexity, ‘practical rationality’ and evidence-based policy making, Policy Polit 2006; 34(1): 115–132.

[55] Tones K . Beyond the randomized controlled trial: a case for ‘judicial review’. Health Educ Res. 1997; 12(2): i–iv. 10.1093/her/12.2.161 10168569

[56] Aaltio I Heilmann P . Case study as a methodological approach. In: Mills A Durepos G Wiebe E (ed.) Encyclopedia of case study research. London: Sage, 2009, pp. 66–76.

[57] Vijay D Zaman S Clark D . Translation of a community palliative care intervention: experience from West Bengal, India. Wellcome Open Res 2018; 3: 66. 30116790 10.12688/wellcomeopenres.14599.1 PMC6069742

[58] Abdul Azeez E Anbuselvi G . Is the Kerala model of community-based palliative care operations sustainable? Evidence from the field. Indian J Palliat Care 2021; 27(1): 18–22. 34035612 10.4103/IJPC.IJPC_45_20 PMC8121230

[59] Judge K Bauld L . Strong theory, flexible methods: evaluating complex community-based initiatives. Crit Public Health 2001; 11(1): 19–38.

[60] Hanberger A . Multicultural awareness in evaluation: dilemmas and challenges. Evaluation 2010; 16(2): 177–191.

[61] Longhofer J Floersch J . Values in a science of social work: values-informed research and research-informed values. Res Soc Work Pract 2014; 24: 527–534.

[62] Fletcher A Jamal F Moore G , et al. Realist complex intervention science. Evaluation 2016; 22: 286–303. 27478401 10.1177/1356389016652743 PMC4946011

[63] Cloete F Auriacombe C . Revisiting decoloniality for more effective research and evaluation. Afr Eval J 2019; 7(1): a363.

[64] Mitchell J . Ethnographic research: a guide to general conduct. London: Academic Press, 1984.

[65] World Health Assembly. Strengthening of palliative care as a component of comprehensive care throughout the life course (WHA 67.19). Geneva: World Health Assembly, 2014.

[66] Stjernswärd J Foley KM Ferris FD . The public health strategy for palliative care. J Pain Symptom Manage 2007; 33(5): 486–493. 17482035 10.1016/j.jpainsymman.2007.02.016

[67] Abraham A . Impact of community-owned home-based palliative care on quality of life of cancer patients in Kerala. Masters in Public Health Dissertation, Sree Chitra Tirunal Institute for Medical Sciences and Technology, India, 2011.

[68] Jayalakshmi R Chatterjee SC Chatterjee D . End-of-life characteristics of the elderly: an assessment of home-based palliative services in two panchayats of Kerala. Indian J Palliat Care 2016; 22(4): 491–498. 27803573 10.4103/0973-1075.191857 PMC5072243

[69] George PN Ganesh MP Chawak S , et al. Factors associated with choosing the Kerala Model of Palliative Care versus standard care among Indian cancer patients. Indian J Med Paediatr Oncol 2022.

[70] Ghoshal A Joad AK Spruijt O , et al. Situational analysis of the quality of palliative care services across India: a cross-sectional survey. Ecancermedicalscience 2022; 16: 1486. 36819806 10.3332/ecancer.2022.1486 PMC9934966

[71] Leaske C . Framework, principles and recommendations for utilising participatory methodologies in the co-creation and evaluation of public health interventions. Res Involv Engagem 2019; 5: 2. 30652027 10.1186/s40900-018-0136-9 PMC6327557

[72] Innes JE Booher DE . Consensus building and complex adaptive systems: a framework for evaluating collaborative planning. J Am Plann Assoc 1999; 65(4): 412–423.

[73] Schwandt T . Post-normal evaluation? Evaluation 2019; 25: 317–329.

[74] McNall M Foster-Fishman PG . Methods of rapid evaluation, assessment, and appraisal. Am J Eval 2007; 28(2): 151–168.

[75] Vijay D Monin P Kulkarni M . Strangers at the bedside: solidarity-making to address institutionalized infrastructural inequalities. Organ Stud 2023; 1: 12979.

[76] Mol A . Care and its values: good food in the nursing home. In: Mol A Moser I Pols J (eds) Care in practice. Vol. 8. Bielefeld, Germany: transcript Verlag, 2010, pp. 215–234.

[77] Vijay D Monin P . Poisedness for social innovation: the genesis and propagation of community-based palliative care in Kerala (India). M@n@gement 2018; 21(4): 1329.

[78] Back L . Live sociology: social research and its futures. Sociol Rev 2012; 60: 18–39.

[79] Walton-Roberts M . Contextualizing the global nursing care chain: international migration and the status of nursing in Kerala, India. Glob Netw 2012; 12(2): 175–194.

[80] Thresia C . Social inequities and exclusions in Kerala’s ‘egalitarian’ development. Mon Rev 2014; 65(9): 28.

[81] Prajitha KC Subbaraman MR Siddharth Raman SR , et al. Need of community-based palliative care in rural India and factors that influence its sustainability: a comprehensive exploration using qualitative methodology in rural Puducherry, India. Palliat Care Soc Pract 2023; 17: 26323524231196315. 37692560 10.1177/26323524231196315 PMC10486217

[82] Ulahannan S Srinivas P Sreekumar S , et al. COVID-19 and multiple inequalities: the case of a coastal community in Kerala. Econ Pol Wkly 2022; 57(30): 24–27. PMC7614310 36919105

[83] Sreekumar S . Understanding Dalit equity: a critical analysis of primary health care policy discourse of Kerala in the context of ‘Aardram’ mission. Int J Equity Health 2023; 22: 165. 37633913 10.1186/s12939-023-01978-4 PMC10463961

[84] Sallnow L Chenganakkattil S . The role of religious, social and political groups in palliative care in Northern Kerala. Indian J Palliat Care 2005; 11(1): 10.

[85] FRHS. Health monitor report. Foundation for Research in Health System. Bengaluru: FRHS, 2012.

[86] Heller P . Social capital as a product of class mobilization and state intervention: Industrial workers in Kerala, India. World Dev 1996; 24(6): 1055–1071.

[87] Keshri VR Sriram V Baru R . Reforming the regulation of medical education, professionals and practice in India. BMJ Glob Health 2020; 5(8): e002765. 10.1136/bmjgh-2020-002765 PMC7462148 32868269

[88] Rajagopal M Kumar S . A model for delivery of palliative care in India – the Calicut experiment. J Palliat Care 1999; 15(1): 44–49. 10333664

[89] Boltanski L . Love and justice as competences: three essays on the sociology of action. Cambridge: Polity, 2012.

[90] Daniel S Venkateswaran C Sunder P , et al. Responding to palliative care training needs in the Coronavirus disease 2019 era: the context and process of developing and disseminating training resources and guidance for low- and middle-income countries from Kerala, South India. Indian J Palliat Care 2020; 26(Suppl. 1): S8–S16. 10.4103/IJPC.IJPC_131_20 PMC7534997 33088079

[91] Acharya M . In conversation with Dr. M.R. Rajagopal, the father of palliative care in India [Internet], https://ehospice.com/international_posts/in-conversation-with-dr-m-r-rajagopal-the-father-of-palliative-care-in-india/ (2022, accessed 21 November 2023).

[92] Aoun SM Nekolaichuk C . Improving the evidence base in palliative care to inform practice and policy: thinking outside the box. J Pain Symptom Manage 2014; 48(6): 1222–1235. 24727305 10.1016/j.jpainsymman.2014.01.007

[93] Visser C Hadley G Wee B . Reality of evidence-based practice in palliative care. Cancer Biol Med 2015; 12(3): 193–200. 26487964 10.7497/j.issn.2095-3941.2015.0041 PMC4607825

[94] Hui D Arthur J Dalal S , et al. Quality of the supportive and palliative oncology literature: a focused analysis on randomized controlled trials. Support Care Cancer 2012; 20(8): 1779–1785. 21935717 10.1007/s00520-011-1275-9

[95] Gomes B Calanzani N Curiale V , et al. Effectiveness and cost-effectiveness of home palliative care services for adults with advanced illness and their caregivers. Cochrane Database Syst Rev 2013(6): CD007760. 10.1002/14651858.CD007760.pub2 PMC4473359 23744578

[96] Wee B Hadley G Derry S . How useful are systematic reviews for informing palliative care practice? Survey of 25 Cochrane systematic reviews. BMC Palliat Care 2008; 7(1): 13. 18715496 10.1186/1472-684X-7-13 PMC2532992

[97] Aoun SM Kristjanson LJ . Challenging the framework for evidence in palliative care research. Palliat Med 2005; 19(6): 461–465. 16218158 10.1191/0269216305pm1057oa

[98] Sackett DL Rosenberg WM . The need for evidence-based medicine. J R Soc Med 1995; 88(11): 620–624. 8544145 10.1177/014107689508801105 PMC1295384

[99] Brownson RC Chriqui JF Stamatakis KA . Understanding evidence-based public health policy. Am J Public Health 2009; 99(9): 1576–1583. 19608941 10.2105/AJPH.2008.156224 PMC2724448

[100] Moore GF Evans RE Hawkins J , et al. From complex social interventions to interventions in complex social systems: future directions and unresolved questions for intervention development and evaluation. Evaluation 2019; 25(1): 23–45. 30705608 10.1177/1356389018803219 PMC6330692

[101] De Silva D Breuer M Lee E , et al. Theory of Change: a theory-driven approach to enhance the Medical Research Council’s framework for complex interventions. Trials 2014; 15: 267. 24996765 10.1186/1745-6215-15-267 PMC4227087

[102] Banerjee D . Provincializing bioethics. Am Ethnol 2022; 49: 318–331.

[103] Iwowo V . Post-colonial theory. In: Coghlan D Brydon-Miller M (eds) The SAGE encyclopedia of action research. London: Sage, 2014, pp. 631–633.

[104] Brown T Bell M . Imperial or postcolonial governance? Dissecting the genealogy of a global public health strategy. Soc Sci Med 2008; 67(10): 1571–1579. 18771835 10.1016/j.socscimed.2008.07.027

[105] Gaotlhobogwe M Major TE Koloi-Keaikitse S , et al. Conceptualizing evaluation in African contexts: Conceptualizing evaluation in African contexts. New Dir Eval 2018; 2018(159): 47–62.

[106] Fives A Canavan J Dolan P . Evaluation study design – a pluralist approach to evidence. Eur Early Child Educ Res J 2017; 25(1): 153–170.

[107] Parry O Gnich W Platt S . Principles in practice: reflections on a ‘postpositivist’ approach to evaluation research. Health Educ Res 2001; 16(2): 215–226. 11345663 10.1093/her/16.2.215

[108] Barclay M Dixon-Woods M Lyratzopoulos G . The problem with composite indicators. BMJ Qual Saf 2019; 28(4): 338–344. 10.1136/bmjqs-2018-007798 PMC6559782 30100565

[109] Atkins L Kelly MP Littleford C , et al. Reversing the pipeline? Implementing public health evidence-based guidance in English local government. Implementation Sci 2017; 12(1): 63. 10.1186/s13012-017-0589-5 PMC5429536 28499393

[110] Clark A . The Mosaic approach and research with young children. In: Lewis M Kellett C Robinson S , et al. (eds) The reality of research with children and young people. London: Sage, 2004, pp. 157–611.

[111] Cram F . Conclusion: lessons about indigenous evaluation. New Dir Eval 2018; 159: 121–133.

